# Iodide Mumps

**DOI:** 10.1097/WOX.0b013e318172fcd4

**Published:** 2008-05-15

**Authors:** Josef Panasoff, David Nusem

**Affiliations:** 1 Allergy Department, Lin Medical, Center Clalit Health Services, Haifa, Israel

## To the Editor

We present 2 cases of bilateral swelling of the parotid glands appearing after application of contrast media. This unusual reaction is also known as "iodide mumps." As there is no premedication that can prevent its appearance, it is advisable in these cases to use imaging systems without iodide-based contrast media.

**Figure 1:**
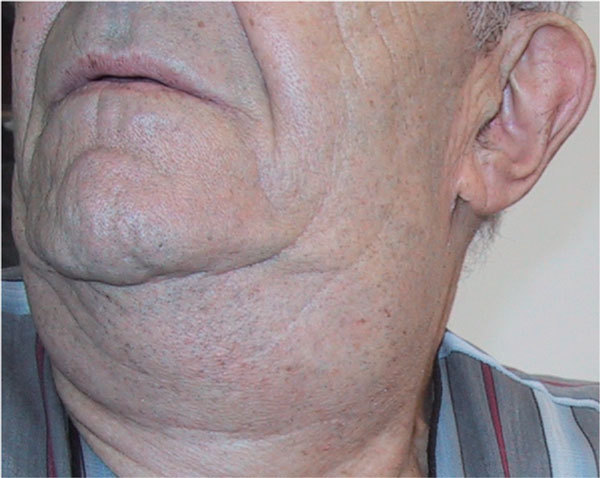
**Swollen parotid gland 24 hours after a lung CT scan with an NICM**.

**Figure 2:**
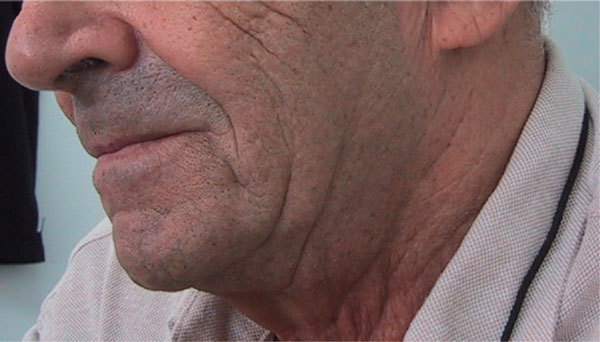
**Five days afterward, the swelling disappeared completely**.

The first case was a 70-year-old man with significant bilateral, painless, cold swelling of the parotid glands (Figure 1). It appeared several hours after performing a lung computed tomographic (CT) scan with 100 mL of iopromide (Ultravist-300; Schering AG), a nonionic contrast medium (NICM). About 5 days after his first visit, the swelling disappeared completely without treatment (Figure 2). Four months before, he had a similar reaction lasting for 5 days, also after performing a lung CT scan with an NICM. Bearing this in mind the second time, he was premedicated with corticosteroids but reacted anyway.

He had adenocarcinoma of the lung on chemotherapy, adult-onset diabetes mellitus, and arterial hypertension treated regularly with glibenclamide and nifedipine. His kidney and liver function tests were normal.

The second case was a 51-year-old man who performed a CT scan with iopamidol (Iopamiro; Bracco SPG). Immediately after the injection, he had a generalized reaction consisting of urticaria and dizziness. He was promptly treated with antihistamines and intravenous steroids, and the reaction subsided. He received 3 more days of oral steroid therapy.

One day afterward, although still on steroids, he developed bilateral swelling of the parotid area. This swelling improved after 1 week and disappeared completely after 2 weeks without any specific treatment. Complete blood cell count and kidney function tests were normal.

Immediate reactions to contrast media are relatively common and considered to be non-IgE-mediated anaphylactoid reactions.

Acute swelling of the submandibular, sublingual, or parotid glands is a rare late reaction to the administration of iodinated contrast media and is referred to as "iodide mumps" or iodide-induced sialoadenitis [1] Ultrasound imaging of the glands shows in these cases diffuse swelling and prominent internal low-echoic septa [2-4].

The cause of this rare complication is not clear. Although compromised renal function has been proposed to be the cause, [5] this can be dismissed in these cases. In a recent article, Gilgen-Anner et al[3] present a case with typical recurrent iodide mumps. Despite an extensive workup, no pathomechanism could be demonstrated, and premedication did not prevent its recurrence.

The occurrence of iodide mumps seems to be related to the capability of the salivary glands to concentrate iodide [6]. The most likely explanation is that for some unknown reason, in a minority of patients, there is an excessive or toxic accumulation of iodide in the ducts of salivary glands leading to its swelling.

Interestingly, in the second case we presented, the patient had both an immediate anaphylactoid reaction and a late iodide mumps-type reaction. This is an unusual combination, rarely happening in the same patient.

Both anaphylactoid reactions and iodide mumps tend to recur, [7] but although premedication with corticosteroids is usually effective in preventing immediate reactions, it does not prevent the occurrence of iodide mumps, as shown above.

It is advisable in these patients to use imaging systems without iodide-based contrast media.

Josef Panasoff, MD

David Nusem, MD

Allergy Department

Lin Medical

Center Clalit Health Services

Haifa, Israel

josefpa@clalit.org.il
